# Characteristics, Management, and In-Hospital Outcomes of Diabetic Patients with Acute Coronary Syndrome in the United Arab Emirates

**DOI:** 10.1100/2012/698597

**Published:** 2012-06-18

**Authors:** Abdulla Shehab, Bayan Al-Dabbagh, Wael Almahmeed, Nazar Bustani, Amrish Agrawal, Afzal Yusufali, Adel Wassef, Abdulla Alnaeemi, Alawi A. Alsheikh-Ali

**Affiliations:** ^1^Department of Internal Medicine, Faculty of Medicine and Health Sciences, United Arab Emirates University, P.O. Box 17666, Al Ain, UAE; ^2^Heart and Vascular Institute, Sheikh Khalifa Medical City, P.O. Box 51900, Abu Dhabi, UAE; ^3^Department of Cardiology, Fujairah Hospital, P.O. Box 10, Fujairah, UAE; ^4^Dubai Heart Centre, Dubai Hospital, P.O. Box 7272, Dubai, UAE; ^5^Obaidallah Hospital (Saif Hospital), MOH, P.O. Box 4727, Ras Al-Khaimah, UAE; ^6^Department of Cardiology, Zayed Military Hospital, P.O. Box 3740, Abu Dhabi, UAE; ^7^Institute for Clinical Research and Health Policy Studies and Tufts University School of Medicine, Boston, MA 02111, USA

## Abstract

We describe the baseline characteristics, management, and in-hospital outcomes of patients in the United Arab Emirates (UAE) with DM admitted with an acute coronary syndrome (ACS) and assess the influence of DM on in-hospital mortality. Data was analyzed from 1697 patients admitted to various hospitals in the UAE with a diagnosis of ACS in 2007 as part of the 1st Gulf RACE (Registry of Acute Coronary Events). Of 1697 patients enrolled, 668 (39.4%) were diabetics. Compared to patients without DM, diabetic patients were more likely to have a past history of coronary artery disease (49.1% versus 30.1%, *P* < 0.001), hypertension (67.2% versus 36%, *P* < 0.001), and prior revascularization (21% versus 11.4%, *P* < 0.001). They experienced more in-hospital recurrent ischemia (8.5% versus 5.1%; *P* = 0.004) and heart failure (20% versus 10%; *P* < 0.001). The mortality rate was 2.7% for diabetics and 1.6% for nondiabetics (*P* = 0.105). After age adjustment, in-hospital mortality increased by 3.5% per year of age (*P* = 0.016). This mortality was significantly higher in females than in males (*P* = 0.04). ACS patients with DM have different clinical characteristics and appear to have poorer outcomes.

## 1. Introduction

The United Arab Emirates (UAE) has the second highest prevalence of diabetes mellitus (DM) in the world, mainly type 2 DM [1, 2]. From a population-based study in the city of Al Ain in the UAE, the age-standardized rates for DM (diagnosed and undiagnosed) and prediabetes among 30–64 year olds were 29% and 24%, respectively [3]. This disease is an expanding health burden globally and particularly in emerging, rapidly developing countries in the Middle East. Because of the proinflammatory and prothrombotic states associated with DM, diabetic patients with acute coronary syndromes (ACSs) are at high risk of subsequent cardiovascular events with poorer outcome and higher mortality rates [4–6]. Patients with diabetes are more likely to experience acute myocardial infarction (AMI) and heart failure. Furthermore, they are at greater risk for dying after an acute cardiac event than patients without DM [7, 8]. However, the management of acute coronary syndromes (ACS) does not differ for patients with diabetes versus without diabetes [9, 10].

The poor prognosis associated with diabetes after acute myocardial infarction (AMI) has been observed in some studies in spite of adjustment for age [11, 12], sex [13], additional comorbidities [14], and coronary risk factors [15]. The presence of diabetes mellitus worsens prognosis in acute coronary syndromes. There is abundant evidence that the prognosis of ACS among diabetic patients is poorer than among nondiabetics [16].

The purpose of this study was to describe differences in the presenting characteristics, management, and hospital outcomes of diabetic and nondiabetic patients with ACS in the UAE using data from the Gulf Registry of Acute Coronary Events (Gulf RACE).

## 2. Subjects, Materials, and Methods

### 2.1. Study Design

The population for our study was derived from the Gulf Registry of Acute Coronary Events (Gulf RACE), a prospective, multinational multicentre registry of patients above 18 years of age hospitalized with the final diagnosis of ACS from various hospitals in 6 Middle Eastern countries. There were no exclusion criteria. Details of the Gulf RACE design and data elements have been described previously [17]. Recruitment in the pilot phase started from May 8 to June 6, 2006. Enrollment in the next phase of the registry started from January 29 through June 29, 2007. Our analysis included 1697 patients hospitalized with an ACS, with and without diabetes mellitus in 18 hospitals in the UAE during this period. These hospitals care for more than 85% of patients with ACS in the UAE. The ACS patients were stratified into those with and without DM. Patients were classified as having diabetes based on the review of medical records and known history of type 1 or type 2 DM treated with diet control, oral hypoglycemic agents, or insulin. Demographic and other baseline clinical characteristics of the patients along with in-hospital management were evaluated. Outcome parameters evaluated during the hospitalization included in-hospital mortality, recurrent ischemia/reinfarction, heart failure, cardiogenic shock, major bleed, stroke, and ventilator requirement. Institutional review board approval or equivalent at each participating hospital was obtained.

### 2.2. Statistical Analysis

Data were analyzed using SPSS version 18 (SPSS Inc., Chicago, IL, USA). Standard descriptive statistics were used. For categorical variables, counts and percentages were reported. Differences between groups were analyzed using Pearson's *X* ^2^ tests (or Fisher's exact tests for cells less than 10). For continuous variables, means and standard deviations were presented, and analyses were conducted using Student's *t*-test. To obtain age- and gender-adjusted in-hospital mortality, the analysis was performed using multivariable logistic regression, and the results were presented as odds ratio (OR) with the associated 95% confidence interval. In all cases, the statistical significance level was set at *P* < 0.05.

## 3. Results

The baseline clinical characteristics, demographics, and risk factors are listed in Table 1. From the 1697 patients enrolled with an ACS, 668 (39.4%) had diabetes, whereas 1029 (60.6%) did not. Patients with diabetes who had an ACS were older, with a mean age of 55.6 ± 11.2 years compared to 50.5 ± 11.5 years for nondiabetics (*P* < 0.001). There was a higher proportion of females in the diabetic group (19% compared to 8.2%, *P* < 0.001). Furthermore, ACS patients with diabetes had a significantly higher body mass index than those without diabetes and were more likely to have a prior history of hypertension, hyperlipidemia, coronary artery disease, coronary artery revascularization, stroke, or peripheral vascular disease, but were less likely to be current smokers (Table 1). Atypical chest pain and dyspnea as the predominant presenting symptoms were more common in diabetic patients when compared to nondiabetics. Diabetic patients with ACS were less likely to present with an STEMI and more likely to have an NSTE-ACS compared to patients without diabetes. During hospitalization, those with diabetes were more likely to have Killip class II–IV heart failure and renal impairment.

Table 2 demonstrates the in-hospital management of the ACS patients. Both groups received aspirin, clopidogrel, heparin, beta-blockers, thrombolytics, and lipid-lowering agents. 194 patients (29.1%) in the diabetic group and 334 patients (32.5%) in the nondiabetic group underwent coronary angiography (CAG). However, patients with diabetes were more likely to be treated with glycoprotein (GP) IIb/IIIa antagonists, angiotensin-converting enzyme (ACE) inhibitors, or beta-blockers (BBs).

Compared with nondiabetics, the group with diabetes experienced more recurrent ischemia, heart failure, cardiogenic shock, and ventilator requirement while in the hospital (Table 3). Mortality was numerically higher among the DM group compared with the non-DM group (2.7% versus 1.6%), but the difference in univariate analysis did not reach statistical significance (*P* = 0.105). Furthermore, diabetes status when evaluated as an independent risk factor for in-hospital mortality in ACS patients with adjustment for age and gender using multivariable logistic regression analysis, remained not statistically significant (OR 1.276, 95% CI 0.63–2.58, *P* = 0.497). From this analysis, the risk of in-hospital mortality increases by 3.5% for every year of age (OR 1.036, 95% CI 1.007–1.066, *P* = 0.016). In addition, our results showed that in-hospital mortality of males was less than half that of females, independent of DM status. This implies that women had a statistically significant adverse in-hospital outcome (death) than men (OR 0.423, 95% CI 0.186–0.962, *P* = 0.04).

## 4. Discussion

We described the baseline characteristics, management, and in-hospital outcomes of patients in the UAE with DM admitted with an acute coronary syndrome (ACS) and the influence of DM on hospital outcomes. Data from recent registries in Europe show that the prevalence of diabetes among ACS patients is rising, ranging from 29 to 35% [18]. Recently, it was shown that 37% of patients with ACS in Oman were diabetic [19]. In the present work carried out in the UAE, diabetic patients represented 39% of the total ACS population studied, and ACS male patients with DM were significantly more compared to females (81% male compared to 19% female). A number of studies and registries have revealed that diabetic patients are at particularly high risk for cardiovascular events. Both GRACE [5] and CRUSADE [20] revealed increased in-hospital mortality in diabetics compared with nondiabetic patients. In this study, diabetic patients who present with ACS were more likely than nondiabetic patients to have a prior angina, myocardial infarction, or stroke. They also had higher BMI, hyperlipidemia, and hypertension [21].

Moreover, as a group, diabetic patients were less likely than nondiabetic patients to be receiving treatment in the hospital with intravenous heparin, although they were more likely to be receiving an ACE inhibitor, a GP inhibitor, or a beta-blocker. GP IIb/IIIa receptor inhibitors, unfractionated heparin or enoxaparin is of proven benefit in diabetic ACS patients [22]. There was no statistically significant difference concerning coronary angiography in patients with diabetes compared to nondiabetics, which is in contrast to previous studies [5, 21]. The use of thrombolytic therapy in STEMI has been examined in numerous studies showing its significant benefit for diabetic patients [23, 24]. In this study, the use of thrombolytic agents in the diabetic group of STEMI patients compared to the nondiabetic STEMI group failed to reach statistical significance (69% compared to 64%, *P* = 0.087).

Hansen et al. [25] observed less use of thrombolytic therapy among diabetic patients compared to nondiabetic patients which could be possibly due to their delayed presentation or yet unverified increased risk of bleeding and atypical symptoms caused by neuropathy [26]. Coronary angiography was not performed on the majority of the patients which is consistent with the results obtained from the full data of the Gulf RACE [17]. There are several factors that could explain the undertreatment of patients with ACS in terms of invasive cardiac procedures, such as the absence or no access to cardiac catheterization labs in the participating hospitals, patients' preferences not to undergo invasive cardiac procedures, and physician's fear of postprocedure complications since most patients were stable with the initial medical treatment.

It is noteworthy to mention that diabetic patients experienced more recurrent ischemia, congestive heart failure, and ventilator requirement while in the hospital (Table 3). Many factors contribute to this adverse outcome in diabetic patients, such as severe diffuse multivessel coronary artery disease, autonomic dysfunction, and diabetic cardiomyopathy [27, 28]. Moreover, increased vascular resistance caused by endothelial dysfunction is not only associated with congestive heart failure but also with diabetes and glucose disturbances in the sub-diabetic range [29]. Finally, in this study, diabetes mellitus was not an independent risk factor for in-hospital mortality, and women had a statistically significant higher adverse outcome (death) than men. This is in accordance with various studies on patients with acute MI reporting that women have higher in-hospital mortality and short-term mortality than men [30, 31]. Similar results have been obtained in studies on gender-based differences in unstable angina or infarction without ST elevation [32].

## 5. Limitations

The strengths of this investigation include its national perspective, the complete spectrum of ACSs experienced by the large number of patients studied, and the use of standardized criteria for defining ACS and hospital outcomes. However, an epidemiologic limitation must be kept in mind when interpreting the results of this study since diabetes is defined as patients known with diabetes, and misclassification cannot be excluded. In particular, diabetes de novo might be misclassified as nondiabetes.

## 6. Conclusion

In conclusion, the results of this national observational study suggest that patients with diabetes in the UAE who develop an ACS have different clinical characteristics and are at risk for adverse nonfatal hospital outcomes, and they appear to represent an important subgroup. Surprisingly DM did not appear to be a high-risk factor for in-hospital mortality. Perhaps, awareness of cardiovascular complication in DM patients may have been a reason for faster more timely and adequate hospitalization. Finally, in view of the very high prevalence of DM in the population, prevention has a great potential for reduction of cardiovascular disease.

## Figures and Tables

**Table 1:** Demographic and other baseline characteristics of the studied subjects (*n* = 1697).

Characteristic	DM (*n* = 668)	Non-DM (*n* = 1029)	*P* value
Age, mean ± SD, years	55.6 ± 11.2	50.5 ± 11.5	<0.001
Female	127 (19)	84 (8.2)	<0.001
Body mass index, mean ± SD, kg/m^2^	27.3 ± 4.4	26.2 ± 4.3	<0.001
Hypertension	449 (67.2)	370 (36)	<0.001
Hyperlipidemia	347 (52)	257 (25)	<0.001
Current smoker (includes sheesha)	266 (40)	557 (54.1)	<0.001
Family history of CAD	84 (12.6)	177 (17.2)	0.01
Prior angina or MI	328 (49.1)	310 (30.1)	<0.001
Past PCI or CABG	140 (21)	117 (11.4)	<0.001
COPD	25 (3.7)	29 (2.8)	0.289
History of stroke	44 (6.6)	29 (2.8)	<0.001
Dialysis	10 (1.5)	7 (0.7)	0.099
PVD	29 (4.3)	16 (1.6)	<0.001

Presenting symptoms			<0.001
Ischemic chest pain	529 (79.2)	908 (88.2)	
Atypical chest pain	45 (6.7)	56 (5.4)	
Dyspnea	70 (10.5)	35 (3.4)	
Palpitation	8 (1.2)	6 (0.6)	
Loss of consciousness	4 (0.6)	7 (0.7)	
Other	12 (1.8)	17 (1.7)	
ST deviation	504 (75.4)	786 (76.4)	0.56

ACS diagnosis			<0.001
Non-STEMI/UA	370 (55.4)	459 (44.6)	
STEMI/LBBB MI	227 (34)	501 (48.7)	
Other	71 (10.6)	69 (6.7)	
Killip class II, III, IV*	182 (27.3)	174 (17)	<0.001
Renal impairment	113 (16.9)	86 (8.4)	<0.001

Figures in parentheses are percentages.

Abbreviations: DM: diabetes mellitus; SD: standard deviation; CAD: coronary artery disease; MI: myocardial infarction; PCI: percutaneous coronary intervention; CABG: coronary artery bypass surgery; COPD: chronic obstructive pulmonary disease; PVD: peripheral vascular disease; STEMI: ST-elevation myocardial infarction; LBBB: left bundle branch block; UA: unstable angina.

*Killip class (scale I–IV) is a risk stratification tool for patients after acute myocardial infarction; a low Killip class indicates a lower likelihood of death within the first 30 days than a high Killip class.

**Table 2:** In-hospital management of acute coronary syndrome patients with and without diabetes mellitus.

In-hospital management	DM (*n* = 668)	Non-DM (*n* = 1029)	*P* value
ASA	655 (98.1)	1023 (99.4)	0.016
CLO	641 (96)	990 (96.2)	0.793
IV HEP	78 (11.7)	175 (17.1)	0.002
LMW HEP	584 (87.4)	886 (86.1)	0.434
GP	288 (43.1)	380 (37)	0.011
BB	440 (65.9)	716 (69.6)	0.102
ACE	466 (69.8)	668 (64.9)	0.031
AIIRB	45 (6.7)	30 (2.9)	<0.001
TX*	153 (69.5)	315 (63.4)	0.087
STA	640 (95.8)	963 (94)	0.051
CAG	194 (29.1)	334 (32.5)	0.273
PCI	100 (15.0)	162 (15.7)	0.681
CABG	45 (6.7)	18 (1.7)	<0.001
IABP	10 (1.5)	11 (1.1)	0.289

Figures in parentheses are percentages.

Abbreviations: DM: diabetes mellitus; ASA: aspirin; CLO: clopidogrel; IV HEP: intravenous heparin; LMW HEP: low molecular weight heparin; GP: glycoprotein IIb/IIIa inhibitors; BB: beta-blockers; ACE: angiotensin-converting enzyme inhibitors; AIIRB: angiotensin II receptor blockers; TX*: thrombolytics for STEMI patients only; STA: statins; CAG: coronary angiography; PCI: percutaneous coronary intervention; CABG: coronary artery bypass graft surgery; IABP: intra-aortic balloon pump.

**Table 3:** In-hospital outcome in acute coronary syndrome diabetic and nondiabetic patients.

Characteristic	DM (*n* = 668)	Non-DM (*n* = 1029)	*P*-value
Recurrent ischemia	57 (8.5)	52 (5.1)	0.004
Infarction	16 (2.4)	26 (2.5)	0.860
Congestive heart failure	134 (20.1)	103 (10)	<0.001
Ventilation	40 (6)	38 (3.7)	0.028
Cardiogenic shock	32 (4.8)	35 (3.4)	0.155
Major bleed	7 (1.1)	8 (0.8)	0.565
Stroke	5 (0.8)	4 (0.4)	0.319
Mortality	18 (2.7)	16 (1.6)	0.105

Figures in parentheses are percentages.

Abbreviations: DM: diabetes mellitus.
